# Self-Assembly Engineering Nanodrugs Composed of Paclitaxel and Curcumin for the Combined Treatment of Triple Negative Breast Cancer

**DOI:** 10.3389/fbioe.2021.747637

**Published:** 2021-08-24

**Authors:** Shuting Zuo, Zhenyu Wang, Xianquan An, Jing Wang, Xiao Zheng, Dan Shao, Yan Zhang

**Affiliations:** ^1^ Department of Breast Surgery, The Second Hospital of Jilin University, Changchun, China; ^2^ Department of Anesthesiology, The Second Hospital of Jilin University, Changchun, China; ^3^ School of Biomedical Sciences and Engineering, South China University of Technology, Guangzhou, China

**Keywords:** triple-negative breast cancer, paclitaxel, curcumin, self-assembly, nanodrug

## Abstract

The clinical outcomes of triple-negative breast cancer (TNBC) chemotherapy are unsatisfactory. Water solubility and biosafety of chemo drugs are also major barriers for achieving satisfactory treatment effect. In this study, we have reported a combinational strategy by self-assembly engineering nanodrugs PC NDs, which were composed of paclitaxel (PTX) and curcumin (Cur), for effective and safe TNBC chemotherapy. PC NDs were prepared through reprecipitation method without using any additional carriers. The PC NDs were preferentially taken up by TNBC cells and we also observed pH-related drug release. Compared with free PTX and simple PTX/Cur mixture, PC NDs have shown higher therapeutic efficiency and better prognosis while the metastasis rate was significantly lower than that of either PTX or PTX/Cur mix group. Therefore, the self-assembly engineered PC NDs might be a promising nanodrugs for efficient and safe TNBC chemotherapy.

## Introduction

Breast cancer is the most common cancer type among women worldwide and threatens their health seriously (McDonald et al., 2016; Yeo and Guan, 2017; Fahad Ullah, 2019). Many chemotherapeutic, hormone-based, and combination drug regimens have been used to treat breast cancer, although patients with advanced aggressive disease (especially triple-negative breast cancer, TNBC) still have poor survival outcomes (Bergin and Loi, 2019). In this context, TNBC is characterized by non-expression of protein receptors, including progesterone receptor, estrogen receptor, and human epidermal growth receptor 2. Relative to other subtypes, TNBC is more aggressive, has a poorer prognosis and higher rates of visceral and central nervous system metastases, with no currently approved targeted therapies (Kumar and Aggarwal, 2016; Akram et al., 2017). Chemotherapy, including neoadjuvant chemotherapy, remains the only standard treatment option for TNBC, although patients have a poor response because of rapidly acquired drug resistance and distant metastasis, and also inevitably experience adverse effects (Lyons, 2019; Yin et al., 2020). Thus, there is a need for combination therapies that can improve the efficacy of current chemotherapeutic strategies for TNBC.

Paclitaxel (PTX) is a first-line chemotherapy drug that is used to treat TNBC by preventing microtubule depolymerization and arresting mitosis at G2/M stages of cell cycle. However, paclitaxel has poor solubility, unavoidable toxicity, and tumors can develop resistance (Bielopolski et al., 2017; Abu Samaan et al., 2019). There is growing evidence that combining PTX with other chemotherapeutic agents (e.g., small molecule inhibitors or natural products) could enhance the anti-tumor effect (Schmid et al., 2018; Kang and Syed, 2020; Mittendorf et al., 2020). Curcumin (Cur) is a hydrophobic polyphenol derived from Curcuma longa (the spice turmeric). It exhibits anti-bacterial, anti-inflammatory, and anti-cancer properties. Furthermore, Cur has been proved to have excellent safety and widespread availability with low cost. Previous studies have indicated that Cur can downregulate the NF-kB and PI3K/Akt signaling pathways in cancer cells, which can inhibit cell growth, induce apoptosis, and increase drug sensitization (Hamzehzadeh et al., 2018; Subramaniam et al., 2018; Chen et al., 2019; Ghasemi et al., 2019; Maiti et al., 2019; Zusso et al., 2019; Borges et al., 2020; Keyvani-Ghamsari et al., 2020). In addition, the combination of PTX and Cur provided synergistic anti-cancer effects and eliminated cancer stem cells in TNBC (Baek and Cho, 2017; Calaf et al., 2018; Saghatelyan et al., 2020). However, similar to PTX, Cur has poor solubility and low bioavailability, which has limited its clinical application. Therefore, it would be useful to develop an efficient and safe system that could simultaneously deliver PTX and Cur to treat TNBC.

Significant work has been dedicated to developing drug delivery system that can concurrently deliver PTX and Cur to the tumor site, which might provide improved therapeutic activity and safety. However, most reported carriers have limited loading capacity and there are also concerns regarding their possible toxicity and biodegradation (Hiremath et al., 2019; Li et al., 2019; Zhao et al., 2019; Shao et al., 2020; Xiong et al., 2020; Hu et al., 2021; Liao et al., 2021; Zhang et al., 2021). Carrier-free drug delivery systems are recently developed alternatives that do not rely on inert carriers and thus avoid potential toxicity. Common strategies for preparing carrier-free nanodrugs include nanoprecipitation, thin-film hydration, template-assisted nanoprecipitation, supercritical fluid techniques, spray drying, and wet media milling (Zhang et al., 2018; Zheng et al., 2018; Sun et al., 2019; Zhou et al., 2019). Relative to free drugs, carrier-free nanodrugs have prolonged blood circulation times, better cellular penetration, and greater tumor accumulation (Yang et al., 2019), which has generated interest regarding their clinical applications. Therefore, we developed a carrier-free nanodrug that is composed of PTX and Cur (PC NDs) using a one-pot self-assembly nanoprecipitation method. Physicochemical, optical and drug release properties of PC NDs were characterized, and we then evaluated their effects against TNBC cells *in vitro* and *in vivo*. The results suggest that PC NDs may be a highly effective and safe option for treating TNBC.

## Materials and Methods

### Chemicals and Reagents

Cur (purity: >94%) and sulforhodamine B (SRB) were obtained from Sigma-Aldrich (St. Louis, MO, United States). PTX was purchased from Solarbio Science and Technology Co., Ltd. (Beijing, China). Dulbecco’s Modified Eagle Medium (DMEM), fetal bovine serum (FBS), trypsin and penicillin-streptomycin (10,000 U/ml) were obtained from GIBCO (Carlsbad, CA, United States). Matrigel was purchased from Corning Inc. (Billerica, MA, United States). Hoechst 33,258 and Lysotracker Red were purchased from Thermo Fisher Scientific (Waltham, MA, United States). Assay kits for determing alanine aminotransferase (ALT), aspartate aminotransferase (AST), alkaline phosphatase (ALP), blood urea nitrogen (BUN), and creatinine (CRE) were obtained from Nanjing Jiancheng Bioengineering Institute (Nanjing, Jiangsu, China). All reagents were directly used without any further purification.

### Preparation and Characterization of PC NDs

The PC NDs were prepared using a reprecipitation method. First, Cur and PTX were dissolved in ethyl alcohol to provide solutions with concentrations of 2 mg/ml. Next, 0.4 ml of the PTX solution and 0.1 ml of the Cur solution were quickly added to 4.5 ml of deionized water, vortexed for 1 min and allowed to stand for 15 min to produce PC NDs. Finally, PC NDs were purified *via* ultrafiltration and collected *via* lyophilization, which provided a 4:1 weight ratio (PTX to Cur) after quantified by UV-vis method.

The morphology of PC NDs was inspected by a transmission electron microscope (JEOL, Ltd., Japan) and a scanning electron microscope (FESEM, S4800, Hitachi Co. Ltd., Tokyo, Japan). Fluorescence spectroscopy was performed using a Shimadzu RF-5301 PC spectrophotometer. UV–vis absorption spectra were obtained using a Shimadzu 3100 UV–vis spectrophotometer. Fourier transform infrared (FTIR) spectra were performed with a Nicolet AVATAR 360 FTIR instrument. X-ray powder diffraction (XRD) investigation was carried out on a Rigaku X-ray diffractometer using Cu Kα radiation. A Nano-ZS 90 Nanosizer (Malvern Instruments Ltd., Worcestershire, United Kingdom) was used to determine the size distribution and zeta potential of PC NDs.

### Drug Release

Drug release behavior was evaluated by adding 5 mg of the PC NDs to a dialysis bag (5,000 Da), which was then placed in 50 ml of phosphate-buffered saline solution (PBS, pH: 7.4 or 5.5) on a shaking table at 37°C for 48 h. Supernatant was then collected and the amounts of PTX and Cur were analyzed *via* high-performance liquid chromatography.

### Cell Culture and Uptake

A mouse breast cancer cell line (4T1), a human TNBC cell line (MDA-MB-231), and a non-neoplastic breast cell line (MCF-10A) were purchased from the American Type Culture Collection. All cells were cultured in DMEM with 10% FBS, 100 U/ml penicillin and 100 U/ml streptomycin in a humidified incubator with an atmosphere of 5% CO_2_. Cellular uptake of PC NDs was evaluated after a 3-h incubation with cells, which were then washed with PBS and co-incubated with Lysotracker Red and Hoechst 33,258. Then cells were observed under an Olympus IX71 fluorescence microscope (Olympus Corporation, Tokyo, Japan). Quantification of cellular uptake was conducted through flow cytometry (FACS, Becton Dickinson Biosciences, Franklin Lakes, United States).

### Cytotoxicity

Cells were first seeded into 96-well culture plates at the density of 5,000 cells per well and then cultured overnight for fully attaching. Free Cur and PTX were dissolved deionized water at a concentration of 1 mg/ml, respectively. Next cells were treated with different final concentrations of free PTX, free Cur, PTX/Cur mixture (PTX/Cur mix) or PC NDs. After being treated for 24 or 48 h, cells were subjected to standard SRB assay and absorbance at 540 nm was analyzed using a multifunctional microplate reader. IC_50_ values were calculated by GraphPad Prism software.

### *In vivo* Experiments

All mice were treated in compliance with the Guide for the Care and Use of Laboratory Animals, and all procedures were approved by the Animal Care and Use Committee of Jilin University (China). 50 μL of 4T1 cells (5 × 10^5^) were mixed with 50 μL of matrigel and then orthotopically injected into the second mammary fat pad of female BALB/c mice (six to eight weeks old). Mice were randomly divided into five groups (n = 6) when tumor volume reached approximately 100 mm3, which were treated using saline, PTX (10 mg/kg, intraperitoneally), Cur (2.5 mg/kg, intraperitoneally), PTX/Cur mix (10 mg/kg of PTX and 2.5 mg/kg of Cur, intraperitoneally), or PC NDs (10 mg/kg, intravenously), respectively. All drug treatments were administered every 3 days, and measurements of tumor volume and body weight were performed at the same time. Tumor volume was calculated according to the following formula: volume = 0.5 × (longest dimension) × (shortest dimension)^2^.

After 21 days of treatment, all mice were sacrificed on day 22. Tumors were measured and weighed. Main organs (liver, spleen, kidneys, hearts, and lungs) were collected, fixed and stained using hematoxylin and eosin (H and E) before being photographed. Biosafety was evaluated based on changes in body weight, pathological changes in organs mentioned above and serum biochemistry indexes including ALT, AST, ALP, BUN, and CRE.

### Statistical Analysis

All experiments were performed at least three times and results were exhibited as mean ± standard deviation. Comparison between groups were calculated by Student’s t-test (two groups) or Bonferroni’s post hoc test (three groups or more). Data were analyzed on SPSS software. Differences were considered statistically significant when *p*-values were less than 0.05.

## Results and Discussion

**FIGURE 1:**
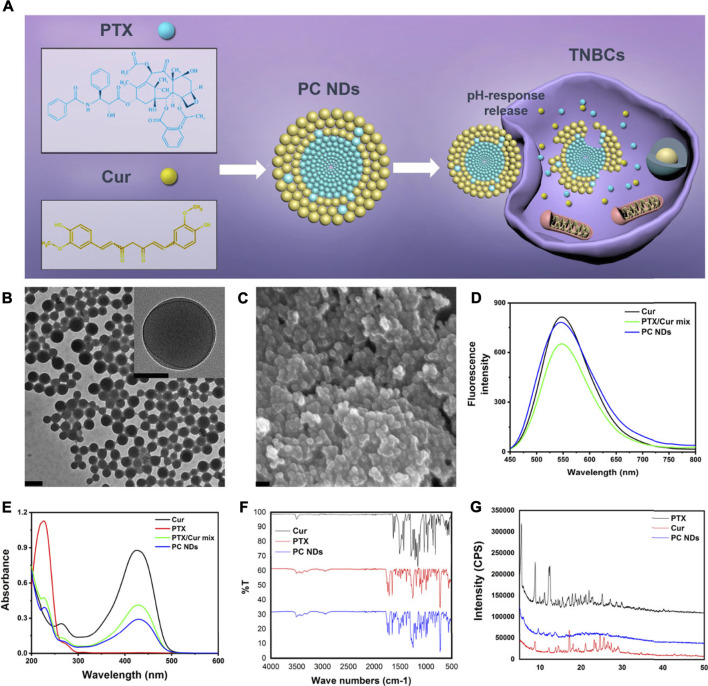
Scheme of PC NDs preparation and its characterization. **(A)** Scheme of PC NDs preparation. PC NDs has been prepared using a reprecipitation method and shown pH-sensitive drug release behavior in tumor cells. **(B)** TEM images of PC NDs. Scale bars are 200 and 50 nm (inset), respectively. **(C)** SEM image of PC NDs. Scale bar is 200 nm. **(D)** Emission spectra of Cur, PTX/Cur mix, and PC NDs. **(E)** UV-vis absorption spectra of Cur, PTX, PTX/Cur mix, and PC NDs. **(F)** FTIR spectra of Cur, PTX, and PC NDs. **(G)** XRD spectra of Cur, PTX, and PC NDs.

The PC NDs were created by quickly adding ethanol solution containing PTX and Cur into excessive volume of deionized water and vortexing the mixture for 1 min (Figure 1A and Supplementary Figure S1). During the nanoprecipitation process, PTX, and Cur molecules were precipitated to form nanoparticles *via* intermolecular interactions, such as hydrogen bonding, π−π stacking and hydrophobic interactions (Li et al., 2018; Hupfer et al., 2021). The PC NDs had a spherical structure (diameter: 120–140 nm) based on characterization *via* TEM and SEM (Figures 1B,C). Zeta potential measurements revealed that the PC NDs had a negative surface charge (−14.6 ± 0.513 mV), which was similar to that of raw Cur (−22.2 ± 3.96 mV) and suggested that the surface of the PC NDs was mostly composed of Cur with phenolic hydroxyl groups. The ultraviolet-visible light absorption and fluorescence spectra of free PTX, free Cur, free PTX/Cur mix, and PC NDs were analyzed. The PC NDs had the same absorption peaks as free PTX and Cur, albeit with variable peak heights (Figures 1D,E). Furthermore, PC NDs have exhibited weaker green fluorescence than free Cur when excited with 420-nm laser, although free Cur, free PTX/Cur, and PC NDs shared the same emission peak (550 nm, Figure 1D). We used FTIR spectra to evaluate whether the bioactive groups of PTX and Cur were preserved in the PC NDs (Figure 1F), which revealed that the PC NDs exhibited N-H stretching vibration at 3,515 cm^−1^ and C=C stretching vibration of the conjugate system in Cur and PTX at 1,513 cm^−1^, as well as disappearance of the strong O-H stretching vibration at 3,511 cm^−1^. Based on these results, we conclude that the PC NDs contained most of the bioactive groups of PTX and Cur, with Cur potentially being “surrounded” by PTX to create the spherical structure of the PC NDs. In addition, we performed XRD measurements to determine whether the PC NDs formed drug eutectics or amorphous formations (Figure 1G), which revealed that the PC NDs had an XRD spectrogram that was similar to that of PTX crystal, with amorphous Cur structures. These findings suggest that the PC NDs were probably composed of crystalline formations containing PTX nanocrystals and Cur.

**FIGURE 2:**
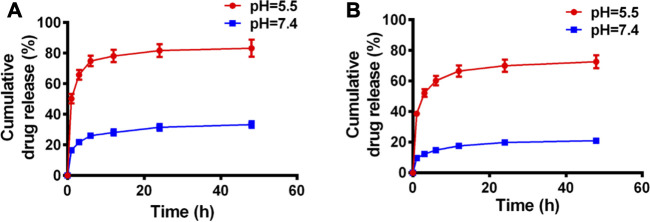
Drug release profiles of PC NDs in PBS of different pH values during 48 h **(A)** PTX and **(B)** Cur release of PC NDs in neutral and acidic PBS.

**FIGURE 3:**
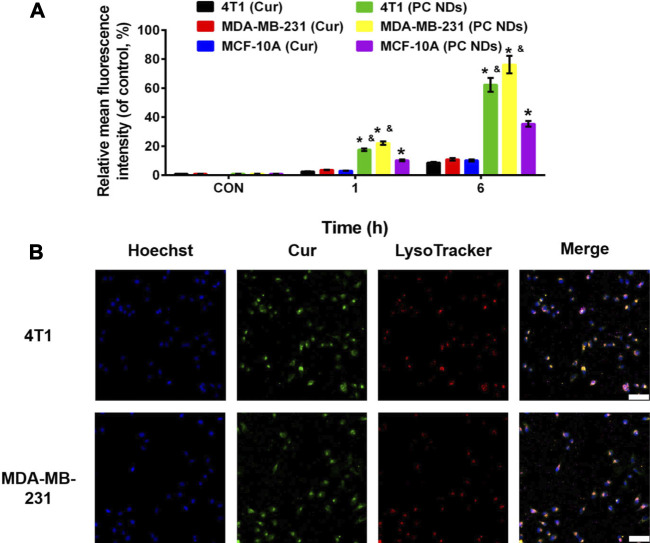
Cellular uptake of PC NDs in 4T1, MDA-MB-231, and MCF-10A cells. **(A)** Quantitative analysis of the internalization of Cur and PC NDs in 4T1, MDA-MB-231, and MCF-10A cells through FACS. Statistical significance: *p < 0.05 vs control and *p* < 0.05 vs Cur.**(B)** CLSM images of 4T1 and MDA-MB-231 cells after incubation with PC NDs for 2 h. Scale bars are 200 μm.

The release profiles of PTX and Cur from PC NDs were evaluated using a dialysis bag and PBS solution, with a pH value of 7.4 to mimic normal conditions in bodily fluids or a pH value of 5.5 to mimic the acidic tumor microenvironment, respectively. As shown in Figure 2A, PTX release showed a time-dependent behavior and reached approximately 33.3% over a 48-h period at a pH of 7.4. However, when the pH was 5.5, PTX release increased substantially and reached 83.2% over a 48-h period. Similar trends were observed in the acid- and time-dependent release of Cur (Figure 2B). Thus, the PC NDs might facilitate simultaneous and preferred release of PTX and Cur in the acidic tumor microenvironment. As shown in Supplementary Figure S2, PC NDs disintegrated quickly in acidic PBS, while the hydration radius has increased by about 25% during 72 h in neutral PBS. Therefore, PCNDs were used just after preparation. Fluorescence microscopy was subsequently used to evaluate cellular uptake of the two drugs. After incubation of 4T1 and MDA-MB-231 cells with PC NDs for 2 h, we labelled the nuclei with Hoechst dye (blue) and lysosomes with LysoTracker RED DND (red). Green fluorescence (Cur) was observed in the cytoplasm and lysosomes (Figure 3B and Supplementary Figure S3), and the overlap of the green and red fluorescence signals suggested that the PC NDs were accumulated in lysosomes. Furthermore, the intensity of the PC ND staining increased with incubation time in the TNBC cells (Figure 3A), which suggested a time-dependent drug release mechanism. As expected, the PC NDs had stronger fluorescence intensity (vs free Cur), which confirmed their higher rate of cellular uptake. However, relative to in the cancer cells, there was significantly less Cur uptake into normal breast cells (MCF-10A), which is likely related to the higher pH in normal cells. When considered together, these findings indicate that PC NDs can be taken up by TNBC cells and their contents released in a pH-dependent manner.

**FIGURE 4:**
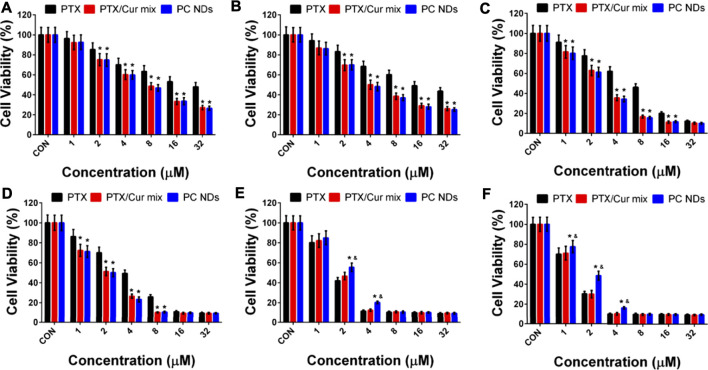
Cytotoxicity of PTX, PTX/Cur mix, and PC NDs in 4T1, MDA-MB-231 and MCF-10A cells after 24 and 48 h incubation. Cell viability of **(A)** 4T1 cells, **(C)** MDA-MB-231 cells, and **(E)** MCD-10A cells after 24 h incubation. Cell viability of **(B)** 4T1 cells, **(D)** MDA-MB-231 cells, and **(F)** MCD-10A cells after 48 h incubation. Statistical significance: *p < 0.05 vs control and *p* < 0.05 vs PTX.

SRB assay was used to determine the viability of 4T1, MDA-MB-231, and MCF-10A cells after 24–48 h of treatment using various concentrations of free PTX, free PTX/Cur mix, and PC NDs. Relative to the control group, 4T1 cell viability of all PTX-containing groups has exhibited a dose- and time-dependent decrease manner (Figures 4A,B), while the addition of Cur provided even greater decreases. Similar results were also observed in MDA-MB-231 cells (Figures 4C,D). At 48 h, the IC_50_ values in 4T1 cells were 16.52 ± 0.16 µM for free PTX, 4.05 ± 0.13 µM for PTX/Cur mix, and 3.87 ± 0.14 µM for PC NDs. Similarly, the IC50 values in MDA-MB-231 cells at 48 h were 7.34 ± 0.19 µM for free PTX, 2.79 ± 0.10 µM for PTX/Cur mix, and 2.58 ± 0.11 µM for PC NDs. Moreover, to our expect, cells incubated with Cur have shown little to no decease on cell viability due to its low concentration (Supplementary Figure S4). These results suggest that combining PTX and Cur provided a significantly greater decrease in TNBC cell viability, relative to PTX alone, which might be related to Cur-induced sensitization of the TNBC cells to PTX (Saha et al., 2012; Yoshida et al., 2017). The small differences between the PTX/Cur mix and PC NDs might be the result of long treatment time, which has given enough time for cells to uptake nearby drugs. It is also worth noting that the PC NDs had less effect on MCF-10A cells than free PTX (Figures 4E,F), which might be related to preferred pH-related drug release in cancer cells (vs in normal cells). When considered together, these results suggest that the PC NDs provided a greater decrease in TNBC cell viability (vs PTX alone), as well as less toxicity in normal cells.

**FIGURE 5:**
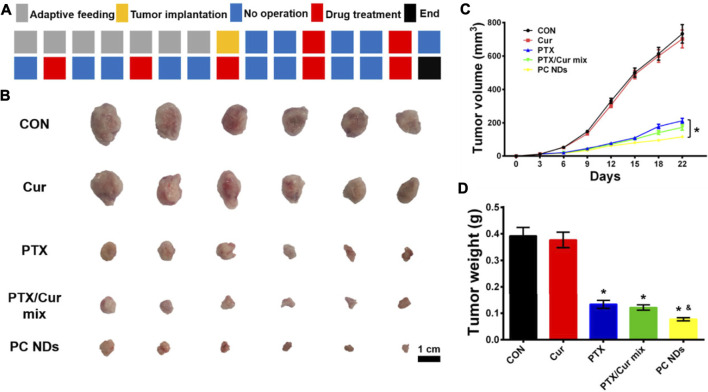
*In vivo* anti-TNBC effect of PC NDs. **(A)** Dosage regimen of treatment for 22 days **(B)** Tumor image, **(C)** Tumor volume, and **(D)** tumor weight. Statistical significance: *p < 0.05 vs PTX in **(C)**; *p < 0.05 vs control and *p* < 0.05 vs PTX in **(D)**.

**FIGURE 6:**
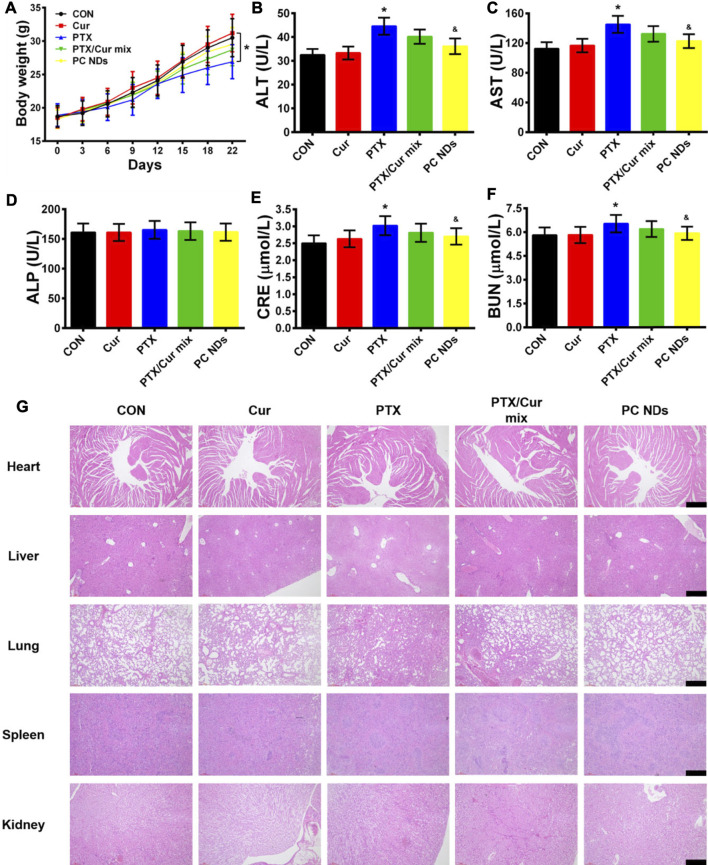
Biosafety profile of PC NDs. **(A)** Body weight, **(B)** ALT, **(C)** AST, **(D)** ALP, **(E)** CRE, **(F)** BUN, and **(G)** H and E staining image of heart, liver, lung, spleen, and kidney from each group. Scale bars are 50 µm. Statistical significance: *p < 0.05 vs control and *p* < 0.05 vs PTX.

The BALB/c mice bearing 4T1 tumors were treated using saline (control group), free Cur, free PTX, free PTX/Cur mix, and PC NDs (Figure 5A). Relative to control group, decreased tumor growth and lower tumor weights were observed at the end of treatment using free PTX, free PTX/Cur mix, and PC NDs (Figures 5B–D), although free Cur did not significantly influence tumor growth. Furthermore, the PC NDs appear to provide substantially more promising results (vs the other formulations), based on a tumor growth inhibition rate of 80.36%. Interestingly, free PTX provided considerable anti-tumor effects, while mice were suffered from a decrease in body weight and abnormal high levels of liver and kidney enzymes (ALT, AST, BUN, and CRE) (Figures 6A–F). In contrast, the PC ND group only exhibited a small decrease in body weight after 21 days of treatment and no histopathological changes in the liver, spleen, kidneys, heart, and lungs (Figures 6A–G) (Edwards et al., 2017; Farhood et al., 2019). Moreover, we observed severe lung metastasis in the PTX and PTX/Cur mix groups, which was not observed in the PC ND group (Supplementary Figure S5) (Sesarman et al., 2018; Tan and Norhaizan, 2019). Thus, co-delivery of PTX and Cur *via* the PC NDs might improve the efficacy of treatment for TNBC (vs free PTX alone), with less systemic toxicity observed in our mouse model, although the underlying mechanisms remain unclear.

In summary, we created biocompatible and carrier-free nanodrugs composed of PTX and Cur *via* a simple nanoprecipitation method. The PC NDs were preferentially taken up by TNBC cells and we also observed pH-related drug release. The cytotoxicity assay revealed that the PC NDs had a greater effect on TNBC cells (vs free PTX), as well as less toxicity in normal cells. The *in vivo* data also clearly indicated that the PC NDs had considerably greater therapeutic efficacy than the free PTX/Cur mixture, with no signs of systemic toxicity. Therefore, the PC NDs might be a promising carrier-free strategy for safely and effectively delivering PTX and Cur to treat TNBC. Further studies are needed to determine whether this nanotherapeutic strategy holds clinical value.

## Data Availability

The raw data supporting the conclusions of this article will be made available by the authors, without undue reservation, to any qualified researcher.
