# Supplementary material for: Self-Assembly Engineering Nanodrugs Composed of Paclitaxel and Curcumin for the Combined Treatment of Triple Negative Breast Cancer
Source: Front Bioeng Biotechnol. 2021 Aug 24;9:747637. doi: 10.3389/fbioe.2021.747637 (PMC8421550; doi:10.3389/fbioe.2021.747637)
Supplement: Supplementary file 1 [file Table1.DOCX]

Supplementary Material for

Self-assembly Engineering Nanodrugs Composed of Paclitaxel and Curcumin for the Combined Treatment of Triple Negative Breast Cancer

**Shuting Zuo^1^, Zhengyu Wang^1^, Xianquan An^2^, Jing Wang^1^, Xiao Zheng^3^, Dan Shao^3, *^, and Yan Zhang^1, *^**

^1^ Department of Breast Surgery, The Second Hospital of Jilin University, Changchun,130041, China;

^2^ Department of anesthesiology, The Second Hospital of Jilin University, Changchun,130041, China;

^3^ School of Biomedical Sciences and Engineering, South China University of Technology, Guangzhou 510006, China.

***Correspondence:**Prof. Yan Zhang, Department of Breast Surgery, The Second Hospital of Jilin University, Changchun,130041, China. E-mail addresses: zhangy01@jlu.edu.cn.

Prof. Dan Shao, School of Biomedical Sciences and Engineering, South China University of Technology, Guangzhou 510006. E-mail: stanauagate@outlook.com.

**
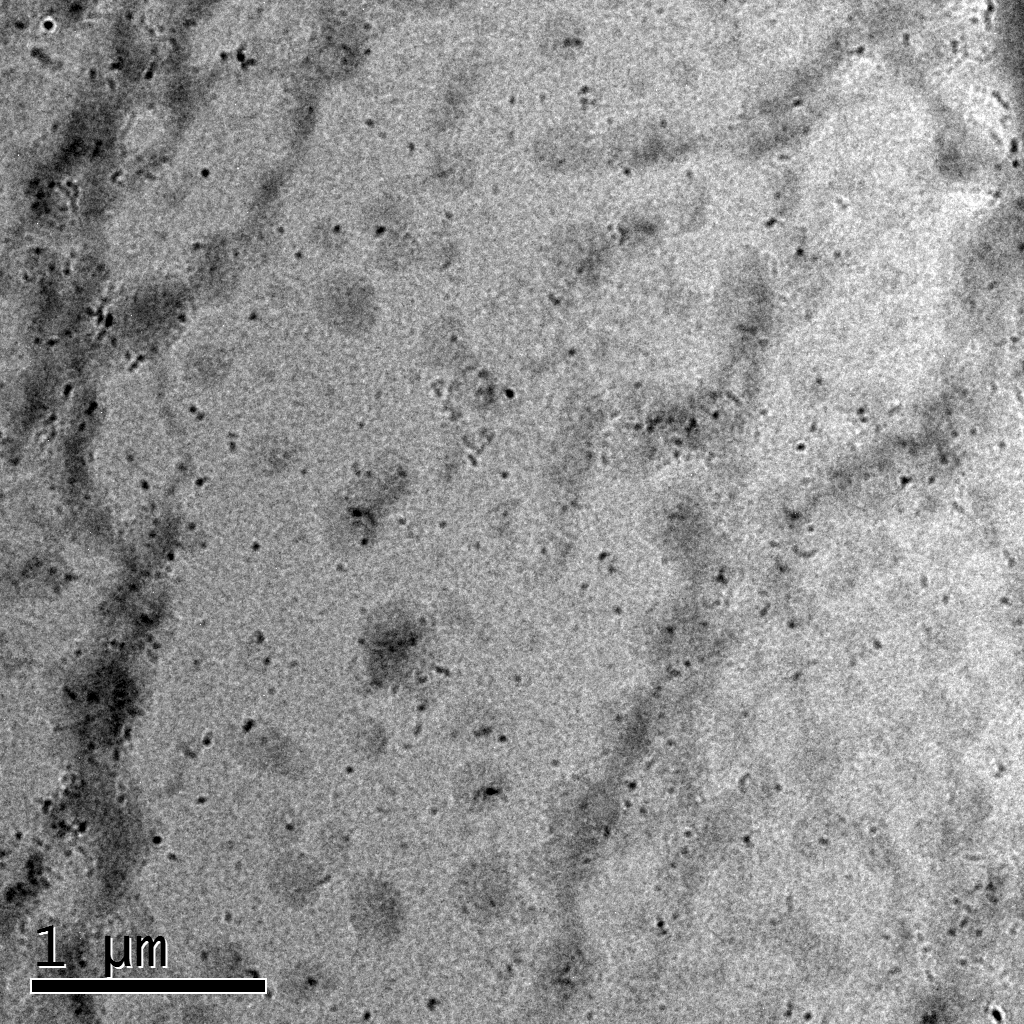
**

**Supplementary Figure 1.** TEM picture of PC-NDs after lyophilized for 6 h.


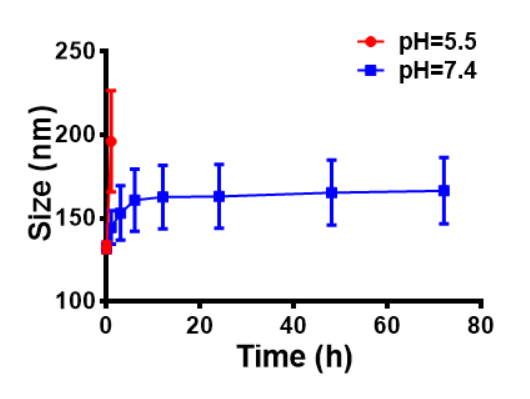


**Supplementary Figure 2.** Stability of PC NDs.


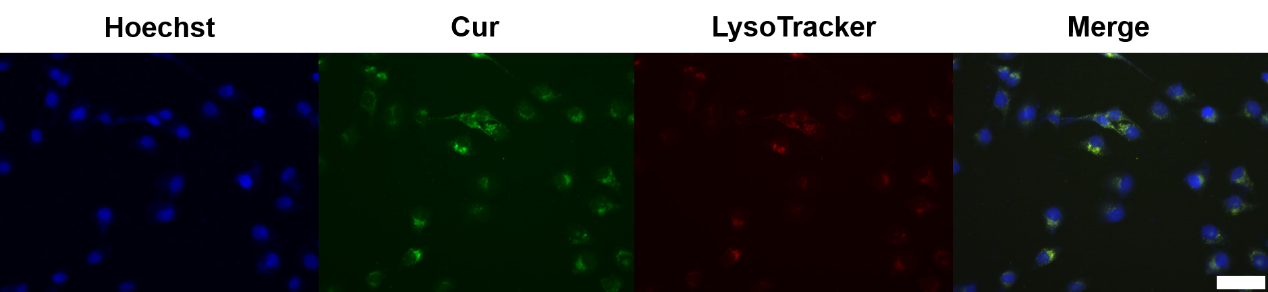


**Supplementary Figure 3.** CLSM images of PC NDs in MDA-MB-231 cells for 2 hours. Scale bar is 200 μm.


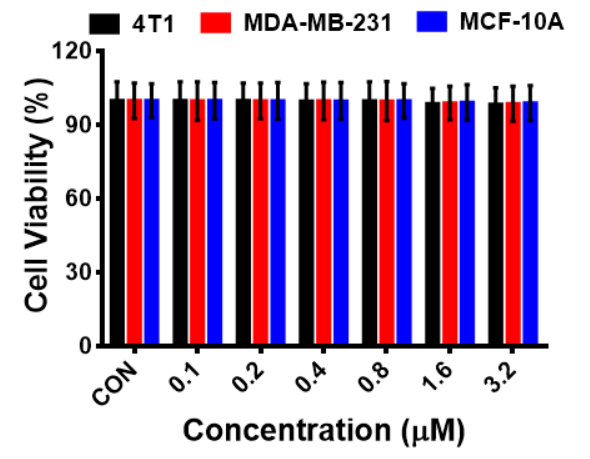


**Supplementary Figure 4.** Cytotoxicity of Cur in 4T1, MDA-MB-231 and MCF-10A cells after 48 h incubation.


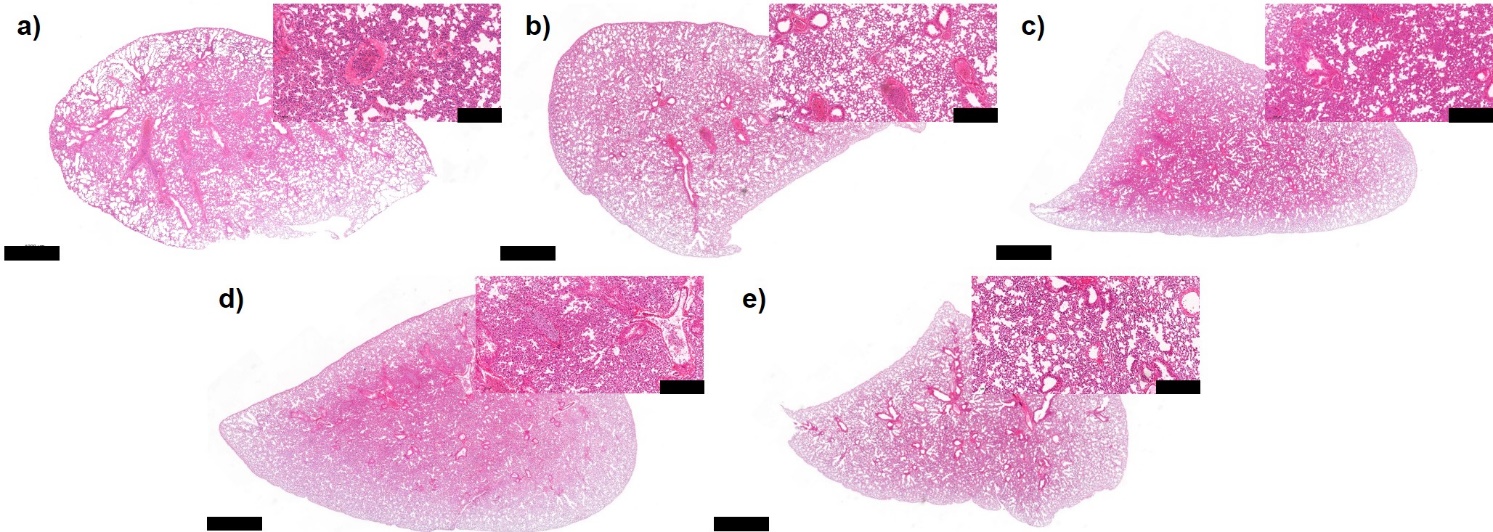


**Supplementary Figure 5.** H/E staining of lungs from a) control, b) Cur, c) PTX, d) PTX/Cur mix and e) PC NDs group, respectively. Scale bars are 1000 µm for pictures of whole lung and 200 µm for enlarged pictures.
